# Lived Experiences of Clinical Depression Among Adolescents With Sickle Cell Anemia in Dar-es-salaam, Tanzania: A Qualitative Study

**DOI:** 10.21203/rs.3.rs-7293105/v1

**Published:** 2025-09-22

**Authors:** Linda Paul Athman, Agnes Jonathan, Fatima Mussa, Honesta John Kipasika, Florence Urio, Mwashungi Ally, Ritha Mutagonda, Emmanuel Balandya

**Affiliations:** Muhimbili University of Health and Allied Sciences; Muhimbili University of Health and Allied Sciences; Muhimbili University of Health and Allied Sciences; Muhimbili University of Health and Allied Sciences; Muhimbili University of Health and Allied Sciences; Muhimbili University of Health and Allied Sciences; Muhimbili University of Health and Allied Sciences; Muhimbili University of Health and Allied Sciences

**Keywords:** Depression, experiences, adolescents, sickle cell anemia, Tanzania

## Abstract

**Background:**

Sickle Cell Anemia (SCA) has been associated with an increased likelihood of neuropsychiatric complications, including depression, and reduced quality of life due to the chronicity of the disease and the occurrence of painful crisis. This study aims to explore the lived experiences of clinical depression among adolescents with SCA in Dar-es-salaam, Tanzania.

**Methodology:**

A qualitative phenomenological study was conducted on adolescents aged 11–19 years clinically diagnosed to have depression who attended sickle cell clinics from October 2023 to March 2024. Experiences of adolescents with depression were obtained through in-depth interviews which were recorded through audio and hand written notes. Themes that were studied and analyzed to ascertain the experience of these adolescents included; health related stigma, acceptance of living with SCA, coping mechanisms to deal with depression and social support.

**Results:**

A total of 6 participants with SCA and depression were interviewed. Majority of adolescents experienced loneliness and isolation and had not fully accepted the reality of living with SCA. Adaptive and maladaptive behaviors were observed as the coping mechanisms when depressed. Social challenges experienced included bullying, name calling and fears of betrayal. Their main sources of support were parents, friends and teachers.

**Conclusion:**

Feelings of loneliness and isolation were commonly experienced by depressed adolescents. Social support, coping mechanisms, and acceptance play pivotal roles in their well-being. Understanding these experiences can inform targeted interventions and support systems for this vulnerable population.

## BACKGROUND

Sickle cell disease (SCD) is a genetic disorder due to a point mutation that results in formation of abnormal hemoglobin molecules which easily polymerize when deoxidized. SCD is a global disease with multisystem effects and various clinical manifestations.^1,2^ Sickle cell Anemia (SCA), which is the commonest genotype with homozygous autosomal recessive hemoglobin S mutation (HbSS), has been linked to the more severe phenotype with significant mortality and morbidity in individuals with African and Mediterranean ancestry.^3^ Tanzania, among other countries, has the highest burden of SCD in East Africa with the estimated prevalence of 8000–11,000 births per year. Approximately half of SCA patients (47.4%) in Tanzania were in the age group of 5–17 years.^4^

Depression is a mood disorder that affects how a person feels, thinks, and conducts daily activities. It has been estimated that 1 in every 7 adolescents experience mental health conditions globally.^5^ The prevalence of depression is estimated to rise with time, increasing disease burden in low and middle income countries.^6^ Majority of depressive disorders arise in adolescents who have experienced long-standing psychosocial difficulties including physical or sexual abuse, emotional abuse, neglect, chronic illness, school difficulties (bullying, academic failure), social isolation, parental psychopathology, family or marital disharmony, and domestic violence.^7,8^

SCA has been associated with an increased likelihood of neuropsychiatric complications, including depression, and reduced quality of life due to the chronicity of the disease and the occurrence of painful crisis. Other factors such as delayed puberty, recurrent hospital admissions and blood transfusions, transition of care to adult clinics and socio economic burden also greatly contribute to depression.^9–10^

Adolescence with both SCA and depression face various challenges in the society including stigma, loneliness, fears of dying, struggles in acceptance and coping with the disease and unavailability of strong social support systems.^11,12^ The huge burden of SCA in Sub-Saharan African countries like Tanzania calls for studies to explore the lived experiences of depressed adolescents with SCA with the goal to inform targeted interventions.

## METHODOLOGY

### Study design and setting

This was a qualitative phenomenological study aimed to explore the lived experiences of clinical depression among adolescents with SCA in Dar-es-salaam, Tanzania. The city is home to four referral hospitals, including Muhimbili National Hospital (MNH) and three Regional Referral Hospitals (RRHs): Mwananyamala RRH, Amana RRH, and Temeke RRH. These hospitals hold weekly sickle cell clinics that provide a range of services, including routine health assessments, pain management, hydroxyurea therapy, vaccinations, infection prevention, complication monitoring, patient and family education, and coordination of specialized care.

### Study participants and sampling

Adolescents aged 11–19 years with moderate to severe depression were obtained through purposive sampling from a database of depressed adolescents with SCA attending sickle cell clinics at Amana, Mwananyamala, Temeke and Muhimbili referral hospitals. Recruited adolescents were previously screened for depression using PHQ-9 tool and channeled to psychologist for further evaluation and management.

### Data collection methods

Subjects were contacted and invited for interviews and prior to interviews consent and assents were obtained. No invited participants declined to participate. In-depth interviews were conducted in convenient places to ensure confidentiality. Interviews lasted 30–45 minutes and were conducted by a pediatric resident trained in qualitative methods. To minimize bias, reflexivity was practiced and themes were reviewed by multiple investigators. Pre-structured open-ended questions were asked in Kiswahili language and enough time was given for the responses. Interviews were conducted until data saturation was achieved. The researcher recorded the interviews using an audio recorder and also took hand written notes.

### Data analysis

Data was transcribed in Swahili language and later translated to English language. Transcribed and translated data were reviewed by the 2 other investigators and thematic analysis was conducted using NVivo II pro (2015). Transcripts were not returned to participants for comment. A three-phase coding process was used: initial open coding, grouping codes into categories, and identifying overarching themes. The coding framework was reviewed by two independent investigators to enhance credibility and ensure consistency. Themes that were studied to ascertain the experience of adolescents living with SCA and depression included; health related stigma, acceptance of living with SCA, coping mechanisms to deal with depression and social support. Interpretation and final write up was made.

## RESULTS

### Qualitative study respondent characteristics.

A total of 6 adolescents (3 males, 3 females) were recruited from those with moderate to severe depression. Those aged 10–14 years accounted for 33.3% while 15–19 years were 66.6% of the study participants. Majority of the participants 83.4% had moderate depression (Table 1).

### Emerging themes

The study uncovered four major themes namely health related stigma, acceptance, copying mechanism and social support systems with subthemes as indicated in Table 2 and discussed hereunder.

### Health related stigma

#### Isolation

Participants demonstrated a sense of isolation which often arouse when individuals were excluded from tasks that others assumed they cannot perform due to their illness. This exclusion was particularly prevalent in school settings and occasionally at home. Such experiences led to strong negative emotions and feelings of inadequacy from the constraints imposed by their illnesses including inability to participate in strenuous sports and chores, which in turn hindered their full participation.

“They engage in activities without informing me, presuming that perhaps I am incapable of participating.”

(Interviewee no 4)

“..People look down on you; seeing that you can’t do anything and they can do everything.”

(Interviewee no 3)

Interestingly, a minority denied experiencing isolation. This could be explained by how their communities perceive their illness and so they neither felt different nor excluded. It may also be because SCA is quite a common disease in this setting, with well-known phenotypic features, so they don’t feel out-casted or singled-out in the community.

“Where I live, sickle cell is considered just like any other disease, so I don’t feel isolated.”

(Interviewee no 3)

#### Loneliness

Loneliness stemming from a lack of connection with others has been closely associated with symptoms of depression. In interviews with adolescents, those who responded expressed individual feelings of loneliness. Some described this experience as being accompanied by unhappiness, worthlessness, and invisibility, often exacerbated by societal judgment.

“Currently, I experience a sense of worthlessness and unhappiness, which contrasts with how I felt initially. The reason behind this emotional shift lies in the impact of my illness or problem…”

(Interviewee no 1)

As a result of experiencing loneliness, significant impact is seen on their emotional well-being. Some reported resultant feelings of being different from others and sometimes absent from their environment, leading to a sense of disconnection and preferably staying in isolated environments.

“….I am not comfortable being around people. I see myself so different from others……I’m just on my own; I don’t have a permanent friend.”

(Interviewee no 5)

“I just like to stay by myself; where I could listen to my music. Most of the times I really like staying alone”

(Interviewee no 4)

#### Acceptance

The majority of adolescents interviewed haven’t fully embraced the reality that they may live with SCA throughout their lives. One interviewee expressed occasional discomfort due to the condition. She continually questions her existence, wondering why she was born with this illness, and imagined an alternate life without it.

“Yes, sometimes it bothers me a lot, for example, I keep asking myself questions like, “Why was I born this way? ‘What would life be like if I were someone else?”

(Interviewee no 4)

Some adolescents struggle to understand what they are experiencing and have yet to fully come to terms with the possibility that they may live with their illness for the duration of their lives. They explore various possibilities, contemplating whether they may access a curative treatment or when they might cease taking medications and achieve full recovery. Despite struggling to comprehend why recurrent painful episodes persist even after treatment, they demonstrate resilience in their efforts to accept this reality.

“I always fail to understand why, even after being discharged from the hospital, you find that the pain returns”

(Interviewee no 1)

“I wonder if this illness will end; I wonder if I will recover or not.”

(Interviewee no 2)

Attitudes toward accepting an illness varied, and this could be influenced by factors such as knowledge on the disease or severity of the disease. During interviews, two adolescents expressed acceptance of their condition and had adapted to living with SCA. Interviewee no. 3 specifically acknowledged her awareness that she would experience periodic painful episodes, which she attributes to the inherent nature of her illness.

“I comprehend that any pain I experience due to sickle cell, whether in my legs, back, or head, is directly linked to the condition. So as a result, it doesn’t significantly trouble me.”

(Interviewee no 3).

#### Coping mechanisms

##### Adaptive coping mechanisms

Majority of adolescents had developed some effective coping mechanisms which were helpful to them based on individual experiences and based on the level of depressive symptoms that they encountered. Some of the repeatedly mentioned coping mechanisms included chatting with friends, watching television or listening to music, staying alone in quiet environment, and meditation. Other coping mechanisms mentioned included ignorance of offending statements, engaging in sports, and avoiding triggering environments.

“I always feel hurt; I would just go home, sit and meditate”

(Interviewee no 1)

“If I feel sad, there is a TV in our house; I will turn on the TV, and watch….if I don’t feel like watching TV, I will go chat with my friends. If not, then I am a tailor; I will take clothes and start sewing.”

(Interviewee no 3)

##### Maladaptive coping mechanism

Adolescents were interviewed to explore their experiences of depressive symptoms and various types of coping mechanisms they have in place when faced with these symptoms. Some adolescents had maladaptive coping strategies following their initial experiences of depressive symptoms. Some adolescents demonstrated anger outbursts and tears while others experienced sadness and low self-esteem. Approximately one-third had developed suicidal thoughts and even attempted self-harm to escape their emotional suffering.

“I said I better die, I told them not to take me to the hospital anymore; I am going to be just fine. I didn’t tell my mother, I stopped taking medicine.”

(Interviewee no 4)

“I wanted to overdose myself. I just found myself in the state of wanting to die……“I took a knife and wanted to cut myself. Another day I took a pen and wanted to pierce myself. Yes, I mean, I take sharp things, the ones that are dangerous and I try to kill myself”……..

(Interviewee no 5)

#### Social support system

##### Sources of social support

Experiences of adolescents with depression and their social support systems were explored. Most of them had reliable social support networks when dealing with depressive symptoms. These networks included parents, friends, and teachers at school. Among the six adolescents interviewed, four mentioned their families, primarily parents, as their primary social support system. Despite overall care, parents served as a source of encouragement. Additionally, one adolescent highlighted a friend who provided social support due to shared experience of having a chronic illness.

“I have one friend who gives me advice. She suffers from epilepsy, and sometimes losses consciousness or falls. She encouraged me to take my medications again.”

(Interviewee no 4)

“I get a lot of comfort from my mother…..I can boldly say that my mother is my pillar of support.”

(Interviewee no 1)

##### Social challenges

Fascinatingly, certain individuals expressed a lack of trust in their friends, fearing potential betrayal and the exposure of their secrets. Some friends even engaged in teasing and bullying behavior. Notably, one adolescent shared that when feeling distressed due to teasing, they confide in their parents or a teacher at school. Only two of the interviewed adolescents had no effective support system

“I can’t stay with one person. You find that today you have this friend, and tomorrow she has another friend. I think she will be sharing your information with other people”

(Interviewee no 5)

When I went back to school, people kept saying, “She has already lost blood, and she was given blood again. When the blood runs out, she will be given cow’s blood”.

(Interviewee no 3)

## DISCUSSION

This study aimed to explore lived experiences of adolescents with SCA and depression. Majority of adolescents with SCA and depression reported to go through health related stigma. They reported to feel isolated and lonely as they were excluded in tasks due to disease limitations. Acceptance of the disease, adaptive copying mechanisms and support from family, friends and teachers were major sources of comfort. Employing strategies to alleviate loneliness and social isolation could improve the mental health of adolescents with SCA who have depression.

In our study, loneliness and isolation were the most common depressive symptoms among adolescents with SCA. This is similar to a cross-sectional study done in the Denmark by Christiansen in 2021 which demonstrated that feelings of loneliness and social isolation were strongly associated with mental illness and depressive symptoms among adolescents and young adults.^12^

Results also reveal that majority of adolescents have not fully accepted the fact that they may have to live with SCA for the rest of their lives. This was similar to findings from Christiansen where adolescents were struggling to understand the pain they go through due to their illness.^12^ Attitudes toward accepting an illness could be influenced by factors such as the knowledge on the disease or severity of the disease. Acceptance of living with an illness has a protective role in improving mental health outcomes for people living with a chronic illness like SCA.^13^

Furthermore, this study revealed that adolescents try to cope with challenges of living with the disease through meditation and distractions such as watching television and music and chatting with friends. Some adolescents were found to develop maladaptive coping strategies like trying to attempt suicide, stop taking their medications and self-harm. A descriptive qualitative study done in Jamaica by Forrester also reported that prayer and spiritual activities were among the major coping mechanism in dealing with the illness, alongside other diversion activities such as watching television, surfing the internet and talking to others.^11^ Good coping behaviors can reduce the chances of developing depression and anxiety disorders in adolescents.

Adolescents with SCA who have developed depressive symptoms experience various challenges in their day-to-day lives. Challenges arising from the society included bullying and name calling among others halting them from developing sincere and comforting relationships. Social support provided by individuals within a close social network significantly improves the overall sense of well-being and aid in coping with chronic illnesses. Main sources of support for adolescents in this study were parents, friends and teachers. Similarly, Forrester reported that strong family, school, and peer support made adolescents with SCA in Jamaica feel accepted in their communities despite their illness.^11^ Having a good social support system could be beneficial to these adolescents who are not only battling this lifelong illness but also going through puberty and hormonal changes that could make them vulnerable to feelings of hopelessness and depression.

## CONCLUSION

Our study has uncovered emotional complexities faced by depressed adolescents living with SCA in Tanzania. Loneliness and isolation were commonly experienced. Social support, coping mechanisms, and acceptance played pivotal roles in their well-being. Understanding these experiences can inform targeted interventions and support systems for this vulnerable population. We recommend routine screening for depression among adolescents with SCA and interventions which should focus on good coping strategies, acceptance of living with SCA and strengthening social support systems.

## Figures and Tables

**Table 1:** Characteristics of the study participants

Adolescents interviewed	n (%)
Age in years	
10–14	2 (33.3)
15–19	4 (66.6)
Gender	
Male	3 (50)
Female	3 (50)
Depression status	
Moderate depression	5 (83.4)
Severe depression	1 (16.6)

**Table 2:** Summary of themes and subthemes studied

THEMES	SUBTHEMES
Health related stigma	1) Isolation
	2) Loneliness
Acceptance	
Coping mechanisms	1) Adaptive coping mechanism
	2) Maladaptive coping
Social support system	1) Sources of social support
	2) Social challenges
